# Unravelling the links between seismo-acoustic signals and eruptive parameters: Etna lava fountain case study

**DOI:** 10.1038/s41598-019-52576-w

**Published:** 2019-11-11

**Authors:** Mariangela Sciotto, Andrea Cannata, Michele Prestifilippo, Simona Scollo, David Fee, Eugenio Privitera

**Affiliations:** 1 Istituto Nazionale di Geofisica e Vulcanologia, Osservatorio Etneo, Piazza Roma 2, 95125 Catania, Italy; 2 Università degli Studi di Catania, Dipartimento di Scienze Biologiche, Geologiche e Ambientali – Sezione di Scienze della Terra, Corso Italia 57, I-95129 Catania, Italy; 3 Alaska Volcano Observatory, University of Alaska Fairbanks Geophysical Institute, Fairbanks, AK 99775 USA

**Keywords:** Geophysics, Seismology, Volcanology

## Abstract

Deriving eruption source parameters from geophysical data is critical for volcano hazard mitigation, yet remains a challenging task in most volcanoes worldwide. In this work, we explored the temporal relationship between geophysical signals and eruptive parameters measured during six explosive episodes from the New South-East Crater of Mt. Etna (Italy). The quadratic reduced seismic velocity and pressure were calculated to track the temporal variation of volcanic elastic radiation, and the lava fountain height was estimated by thermal camera image processing. The temporal relationships between these geophysical and eruptive time series were studied. In particular, the first considered lava fountain exhibited a “clockwise hysteresis” pattern: higher seismic amplitude with respect to the fountain height during the waxing phase as compared to the waning phase. We also calculated the regression parameters for both linear and power laws, linking seismo-acoustic and eruptive time series. For the linear regressions, we found fairly constant values of the scaling factors in five out of six eruptive episodes, which can be considered as a promising step to derive eruption source parameters from geophysical data in real-time. Regarding power law regressions, a clear relationship was observed between the exponents determined for the power law linking quadratic reduced velocity and lava fountain height, and the time interval duration from the previous eruption. These results suggest that the condition of the uppermost part of the plumbing system (e.g. viscosity of residing magma and conduit conditions) play a key role in the seismic energy generation during the eruptions.

## Introduction

Effective hazard assessment and consequently risk mitigation during ash-rich explosive activities mainly rely on volcanic ash transport and dispersion models, which require as input the eruption source parameters (i.e. mass eruption rate, column height, total mass), that can change rapidly during an eruption (e.g.^1,2^). However, on the basis of the current state of scientific knowledge, real-time estimation of the eruption source parameters is not feasible for most volcanoes worldwide (e.g.^1,3^). Indeed, the real-time retrievals of those parameters can be carried out with remote sensing systems as radars and lidars that, however, are expensive and used at only a few volcanoes (e.g.^3–6^). Seismic and infrasonic monitoring is routinely operated on many volcanoes around the world and sensors are relatively cheap. Hence, deriving eruption source parameters from real-time streaming of these geophysical data is a fascinating and challenging task that could seriously improve the monitoring of ash-rich explosive eruptions.

Some studies have shown how the increase in seismic amplitudes (for instance due to volcanic tremor variations) corresponds to the intensification in magma discharge rate, column height and, consequently, to the Volcanic Explosivity Index (e.g.^7–12^). Furthermore, infrasound has been used to infer mass eruption rate, plume height and volatile mass flux (e.g.^11,13–17^).

As summarized by Ichihara^11^ who focused on eruption seismic and acoustic tremor, most of the authors have developed power-law models with seismic or acoustic squared-amplitude or energy proportional to eruption rate raised to a particular exponent ($SET\propto \,{\dot{V}}_{m\,}^{a}\,$and $\,AET\propto \,{\dot{V}}_{m\,}^{b}$, where *SET* and *AET* are the seismic ad acoustic power, $\dot{V}\,$the volume flux and *a* and *b* the power law exponents for seismic and acoustic data, respectively). The power-law exponents found by previous studies range between 0.9 and 4 for seismic signals and between 3 and 10 for infrasound signals^11^. The exponent variability depends on both theoretical models of seismic and infrasonic tremor sources and observations used to define them. For example, Prejean and Brodsky^18^ defined exponent equal to 4 for the seismic energy vs magma discharge rate relationship by considering a model with a single force applied to the ground by erupting jets. In a different model, McNutt and Nishimura^19^ assumed that the seismic tremor is composed of far-field Rayleigh waves generated by a radially oscillating cylindrical conduit, and found that the tremor amplitude should be proportional to the product of the pressure fluctuation amplitude and the source volume. By studying sub-Plinian events in the 2011 eruption of Shinmoe-dake (Japan), Ichihara^11^ found pairwise linear relationships between seismic energy, infrasonic energy and magma discharge rate, suggesting a common seismic-infrasonic source, located in the subsurface. This source could be successive explosions at the fragmentation surface in the conduit under steady state flow condition. Similar to Ichihara^11^, Haney *et al*.^20^ established analogies between equivalent source theory in seismology and acoustics and, by taking into account a force source model of a volcanic eruption, found a linear scaling between seismic energy and eruption rate. However, they also stated that this relationship is valid only assuming a constant excess pressure within the reservoir and a constant Strouhal number. Changes in the exponent (even during the same eruption) can be due to the fact that the eruptive seismic tremor shifted from a single force source to a source better represented by a moment tensor or vice versa^20^.

**Figure 1:**
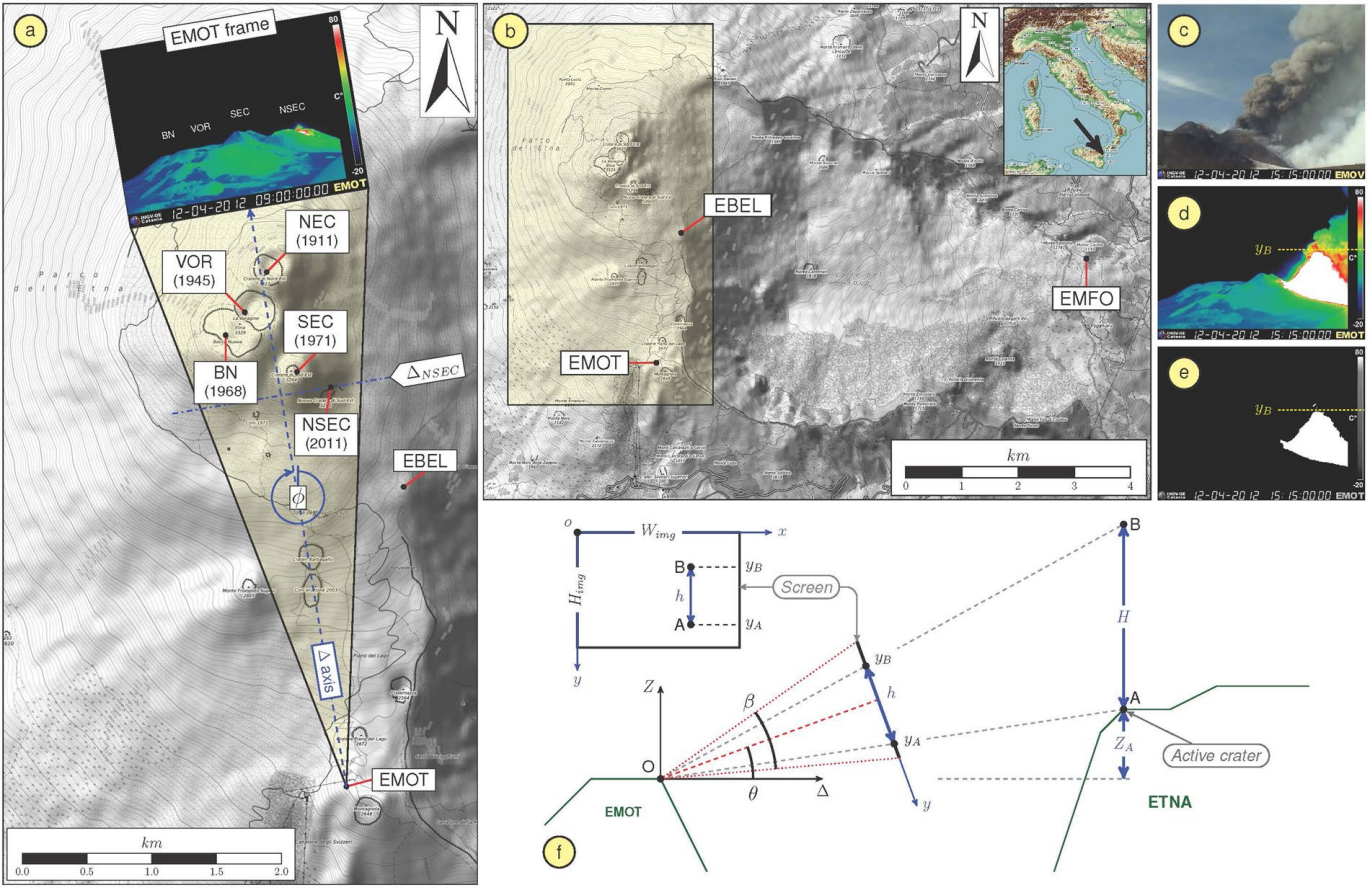
Mt. Etna map and monitoring networks. (**a**) Digital elevation model of the summit area of Mt. Etna with the location of: the summit craters and the corresponding date of origin (South-East Crater: SEC; New South-East Crater: NSEC; Bocca Nuova: BN; Voragine: VOR; and North-East Crater: NEC), the thermal camera EMOT with the related field of view (yellowish triangle area) and an example of recorded frame, and the location of the seismo-acoustic station EBEL. (**b**) Digital elevation model of Mt. Etna with the location of the monitoring video camera EMOT and the seismo-acoustic stations used in this work (EBEL and EMFO). (**c)** Camera field-of-view for EMOV, co-located with the EMOT camera and recording images in visible band. (**d**) Camera field-of-view for EMOT, recording thermal images. (**e**) Binary image obtained by the frame in (**d**) by choosing a temperature threshold. (**f**) Geometry schematic of the camera with respect to the active crater and to the lava fountain. Maps in (a,b) derive from OpenTopoMap.org (https://creativecommons.org/licenses/by-sa/3.0/).

Empirical relationships have also been derived via seismic and infrasound data. Iguchi^12^ found a linear empirical relationship between the monthly sum of ash weights and the monthly sum of the square of seismic tremor amplitudes and ground deformation at Sakurajima Volcano. Eaton *et al*.^21^ and McNutt *et al*.^22^ obtained similar linear relationships between volcanic tremor amplitude and lava fountain height for the 1959 Kilauea Iki eruptions and the 1986 Pavlof eruptions, respectively. Bernard *et al*.^23^ obtained various scaling factors correlating ash fallout mass and quadratic median amplitude of volcanic tremor in order to indirectly estimate eruptive source parameters from geophysical data at Cotopaxi volcano. The empirical equations obtained in most studies are mainly based on a few eruptions that occurred at the same volcano or perhaps at different volcanoes. Furthermore, the volcanological parameters (such as ash weights and height of the plume) describing a single eruption are generally characterized by a low sampling rate when compared to the sampling rate of the seismic and infrasonic time series. In spite of the dense multiparametric monitoring network and the frequent eruptive activities, such a kind of analysis, allowing to estimate quantitative empirical relationships between geophysical and volcanological data, has never been performed at Mt. Etna volcano. Indeed, the frequent activity has produced more than fifty explosive events from summit craters and lateral fissures in the last decade. Furthermore, Etna is monitored by one of the densest multiparametric permanent networks worldwide (i.e., seismic, infrasonic, video; e.g.^24^). The summit area is currently composed of 5 craters (Fig. 1a): South-East Crater (SEC), New South-East Crater (NSEC), Bocca Nuova (BN), Voragine (VOR), and North-East Crater (NEC). Since 2011 the most active crater has been NSEC (e.g.^25^).

In this work, we investigated the relationship between seismo-acoustic signals and eruptive parameters (e.g. lava fountain heights) during six paroxysmal episodes produced by NSEC. We find time-dependent relationships during each eruption and an overall dependence related to inter-eruption intervals. These suggest that the state of the upper conduit strongly influences seismic and acoustic wave generation.

### Eruptive activity between 2011 and 2017

Since January 2011, a great number of explosive and effusive eruptions has taken place at Etna with 25 episodes of lava fountains that occurred at NSEC until April 2012 (e.g.^26^). In 2013, Etna produced 19 lava fountains from NSEC, and numerous short episodes of Strombolian activity from BN, VOR and NSEC^27^. In the following year (from mid-December 2013 to the end of 2014), longer and milder activity at both NSEC and an eruptive fissure opened at the base of NEC during July-August, replaced the previous lava fountains (e.g.^27^). The last significant eruptive episode in 2014 was a lava fountain, which took place at NSEC on 28 December (e.g.^28^). The first few months of 2015 were characterized by Strombolian activity at multiple summit craters, up to the first days of December when four paroxysmal activities occurred from VOR for the first time since the 1998–1999 eruptive events (e.g.^25^). The eruptive activity in May 2016 was characterized by three lower intensity episodes of lava fountains at VOR and in March 2017 from the NSEC.

On the basis of this brief chronology, lava fountains have been the most common eruptive activity at Etna in recent years. These episodes are generally characterized by a similar succession of different phases^26,27^, which can be summarized as: (i) a reactivation phase, when minor intra-crater explosive activity takes place and emits ash; (ii) Strombolian activity, with variable durations from a few hours to several days; (iii) lava emissions; (iv) intensification in explosive activity culminating in a lava fountain characterized by a continuous jet of magma and gas, lasting from a few tens of minutes to several hours; (v) waning phase with lava fountains being gradually replaced by mild Strombolian activity, lasting from a few up to tens of minutes.

In this manuscript, we focus our analyses on six lava fountain events selected on the basis of the quality of seismic, infrasonic and video camera data: 12 January 2011, 10 April 2011, 12 August 2011, 29 August 2011, 4 March 2012 and 12 April 2012. All the events were generated from the NSEC. Detailed description of each fountaining episode is reported in the supplementary information.

## Data and Methods

### Volcanological data acquisition

Etna eruptions are in part monitored by a network of optical and thermal video cameras, located at different distances and azimuths from the summit area and transmitting images in real time to the 24–7 control room in Catania (e.g.^24^). In our study, we focus the analysis on the thermal camera located nearest to the active crater (NSEC), named EMOT (Fig. 1a,b,d). EMOT acquires thermal images with a sampling rate of 1 Hz at a distance of ~3 km from the NSEC.

### Seismic and infrasonic data acquisition

Eruptive activity at Etna produces a large variety of seismic signals, such as volcanic tremor, long period (LP) and very long period (VLP) events, and volcano-tectonic earthquakes (e.g.^24,29^). The study of the first three types of signals, likely related to fluid dynamics inside the plumbing system (e.g.^30^), is typically performed using signals recorded by broadband seismic stations close (within ~10 km) to the summit area. In this work, we used signals recorded by EMFO and EBEL stations (Fig. 1a,b), located ~8 and 1.5 km from the summit, respectively, and equipped with broadband three-component Nanometrics Trillium 40 s seismometers and recording at a sampling rate of 100 Hz.

The acoustic activity of Etna, accompanying both degassing and explosive activity (e.g.^31^), is monitored by a network of 9 infrasonic stations. Each station is equipped with GRASS 40AN microphones, with a flat response in the frequency range 0.3–20,000 Hz, acquiring at a sampling rate of 50 Hz. In this work we used infrasound data from stations EMFO and EBEL stations (Fig. 1a,b), the same stations used in the seismic analysis. The infrasound sensors are located a few m from the seismometers.

### Methods

Analysis of lava fountain height from camera data has previously been used to explore important relationships with geophysical data (e.g.^32–34^). Hence, the temporal evolution of the lava fountain height was estimated using images taken by the EMOT thermal camera (Fig. 1a,d). To obtain the time series of lava fountain heights, we followed a similar approach to Carbone *et al*.^32^. In this method, each frame was converted into a binary image by choosing suitable temperature threshold values. The pixel height of the saturated part of the image (representing the incandescent material ejected during the explosive activity) was calculated from the rim of NSEC (Fig. 1c–e) in successive 1 Hz images. It is important to measure only the portion of eruptive column characterized by momentum-driven jets and neglect the buoyant region. To do that, only a narrow vertical band right above the vent was taken into account^32^. Finally, the height in pixels was converted into meters above the vent using the following steps: (i) the absolute coordinate system is determined from the position of the thermal camera (Fig. 1a); (ii) the coordinates of the summit craters are estimated using UTM (zone 33, WGS84); (iii) the pinhole model (without distortions) has been used for the camera (Fig. 1f)^35,36^; (iv) the intrinsic parameters of the camera have been estimated using the camera calibration toolbox for Matlab (http://www.vision.caltech.edu/bouguetj/calib_doc/)^37^; (v) we assumed that the roll angle of the camera was negligible; (vi) we estimated azimuth (ϕ) (Fig. 1a) and elevation (θ) angles (Fig. 1f) using the correspondence between the position of the summit craters and the relative projection on the frame of the camera; (vii) we developed an image processing tool to estimate the top (${y}_{B}$) of the lava fountain in each frame (Fig. 1e); (viii) the height of the lava fountain was estimated using the following equations:

1 $$\{\begin{array}{l}\,H={\Delta }_{A}\frac{\sin (\theta )+m\,\cos (\theta )}{\cos (\theta )-m\,\sin (\theta )}-{z}_{A}\\ \,m=(1-2\frac{{y}_{B}}{{H}_{img}})\tan (\frac{\beta }{2})\\ \,{\Delta }_{A}={x}_{A}\,\sin (\varphi )+{y}_{A}\,\cos (\varphi )\end{array}$$

where the subscripts *A* and *B* indicate the active crater and the top of the lava fountain, respectively.

The definitions of *z*_*A*_, *x*_*A*_, *y*_*A*_, *y*_*B*_ and *β* are given in Fig. 1f.

**Figure 2:**
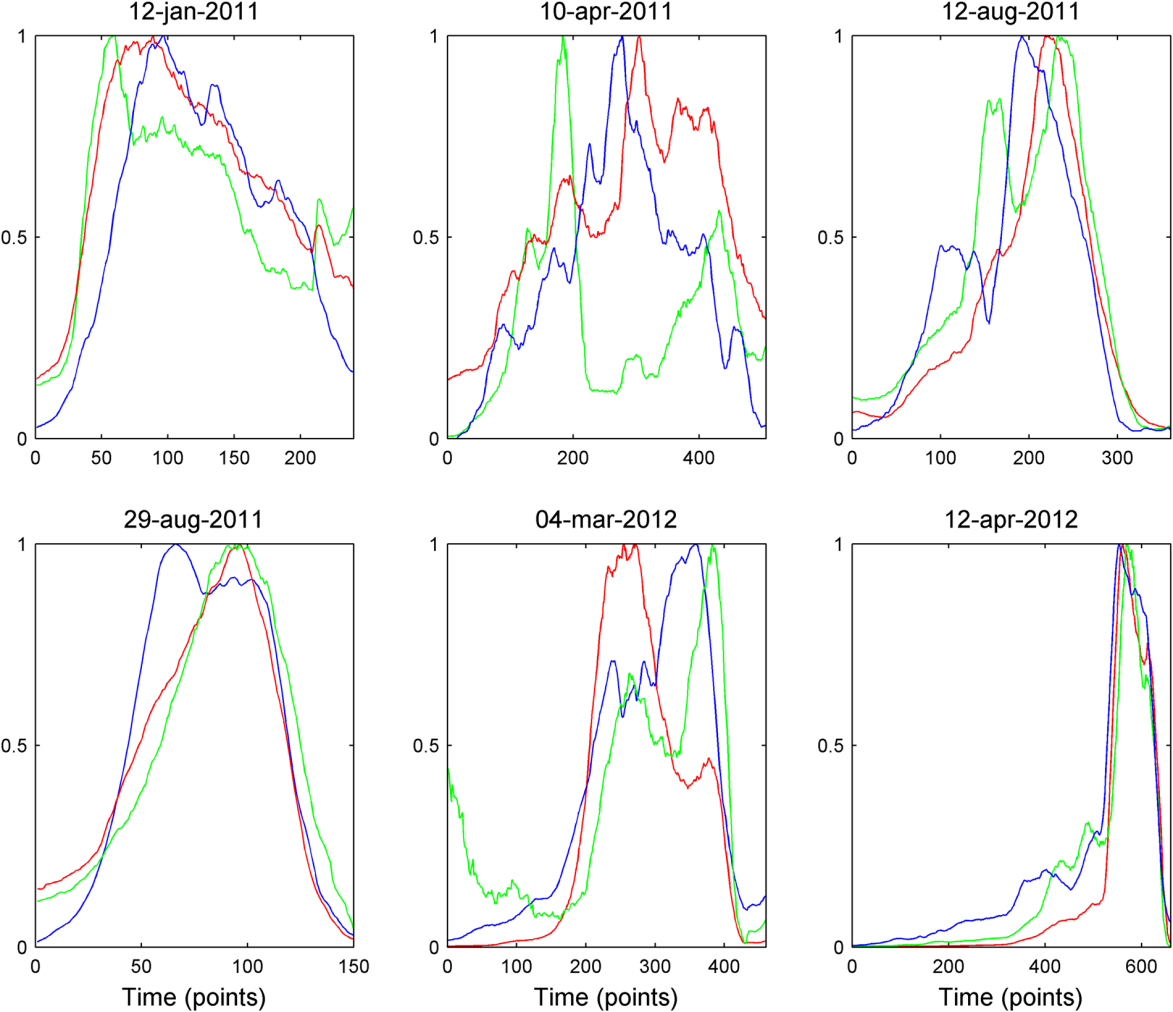
Multiparametric time series. Normalized quadratic reduced velocity (red line) and pressure (green line) envelope in the 0.5–5.0 Hz frequency band, computed at EMFO, and lava fountain height values (blue line) for each eruptive episode.

In order to track the evolution in time of both seismic and infrasonic signals, we used the quadratic reduced velocities and pressures that do not depend on the source-station distances. The quadratic amplitudes were chosen over simple amplitudes to be proportional to the signal energy. As for the source, since the eruptive crater is NSEC, we considered both acoustic and seismic sources located in this crater. This is also confirmed by the location of volcanic tremor centroids, infrasonic events and infrasonic tremor that are displayed in the supplementary information (Fig. S1). The quadratic reduced values were calculated after retrieving the root mean square (RMS) amplitudes of volcanic tremor and infrasound time series and then by multiplying them for the source-station distance. The quadratic reduced velocities and pressures were calculated over 30 sec-long windows without overlapping and in 4 distinct frequency bands (0.5–1.0, 1–2, 2–3 and 0.5–5.0 Hz). Time series of lava fountain height, quadratic reduced velocities and pressures were then smoothed over 15-minute-long windows to facilitate comparisons (Fig. 2).

We set the beginning of the time window used for comparison as the instant when the lava fountain height exceeded a value of 0 m above the crater and the end when it returned to a background level. In such a way, we were able to explore both the most energetic Strombolian activity and the paroxysmal phases of the eruptive episodes.

**Figure 3:**
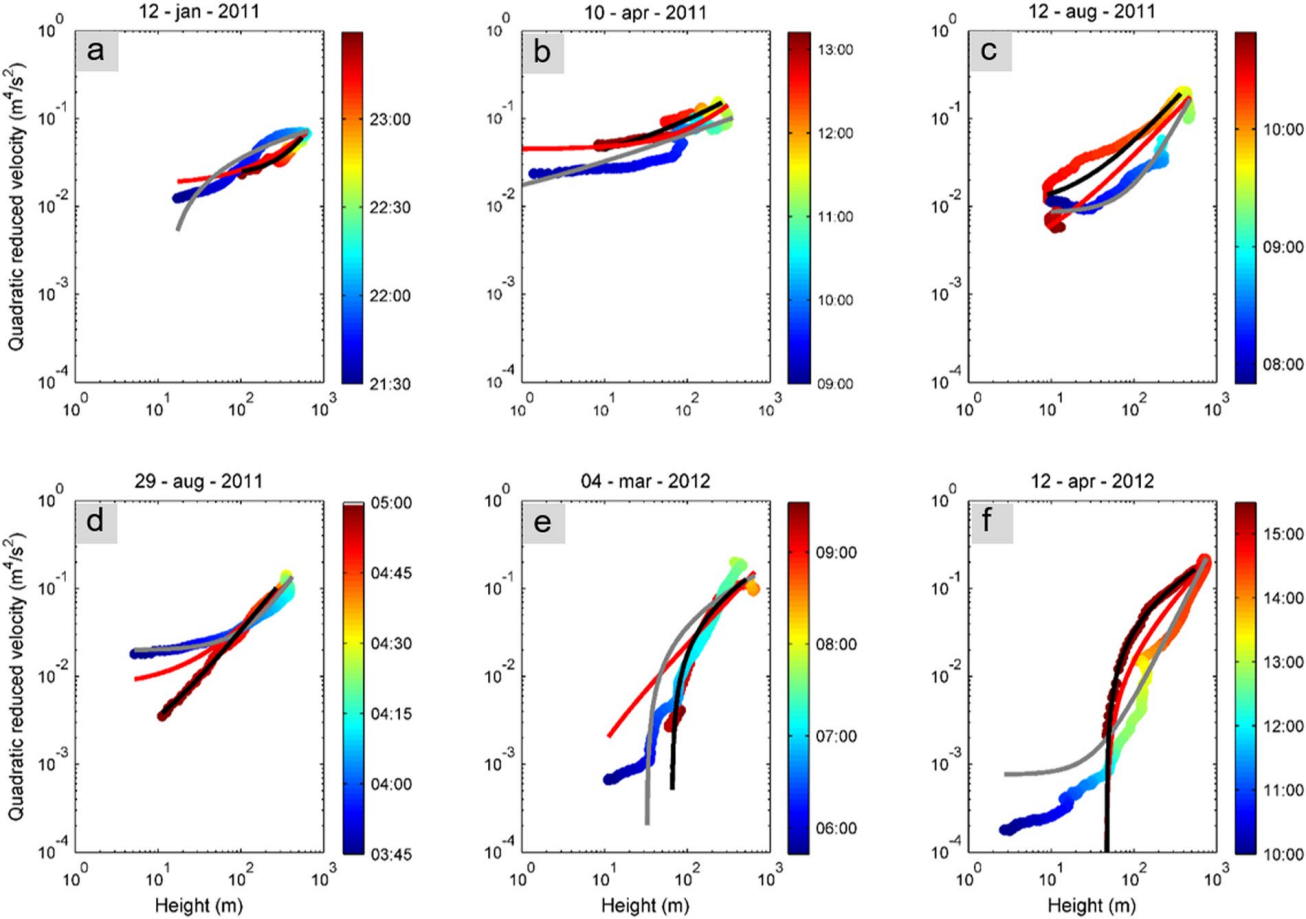
Seismic-lava fountain height relationship. Relationship between quadratic reduced velocity, computed at EMFO, and lava fountain height as a function of time (in color scale) in the 0.5–5.0 Hz frequency band for each eruptive episode. Red curve represents the best fit assuming a linear relationship between the two variables; grey and black curves represent the best fit assuming a power law function, thus with the exponent (α in Eq. 2) variable for the waxing (solid grey) and the waning (solid black) phases of the eruptive episode.

**Figure 4:**
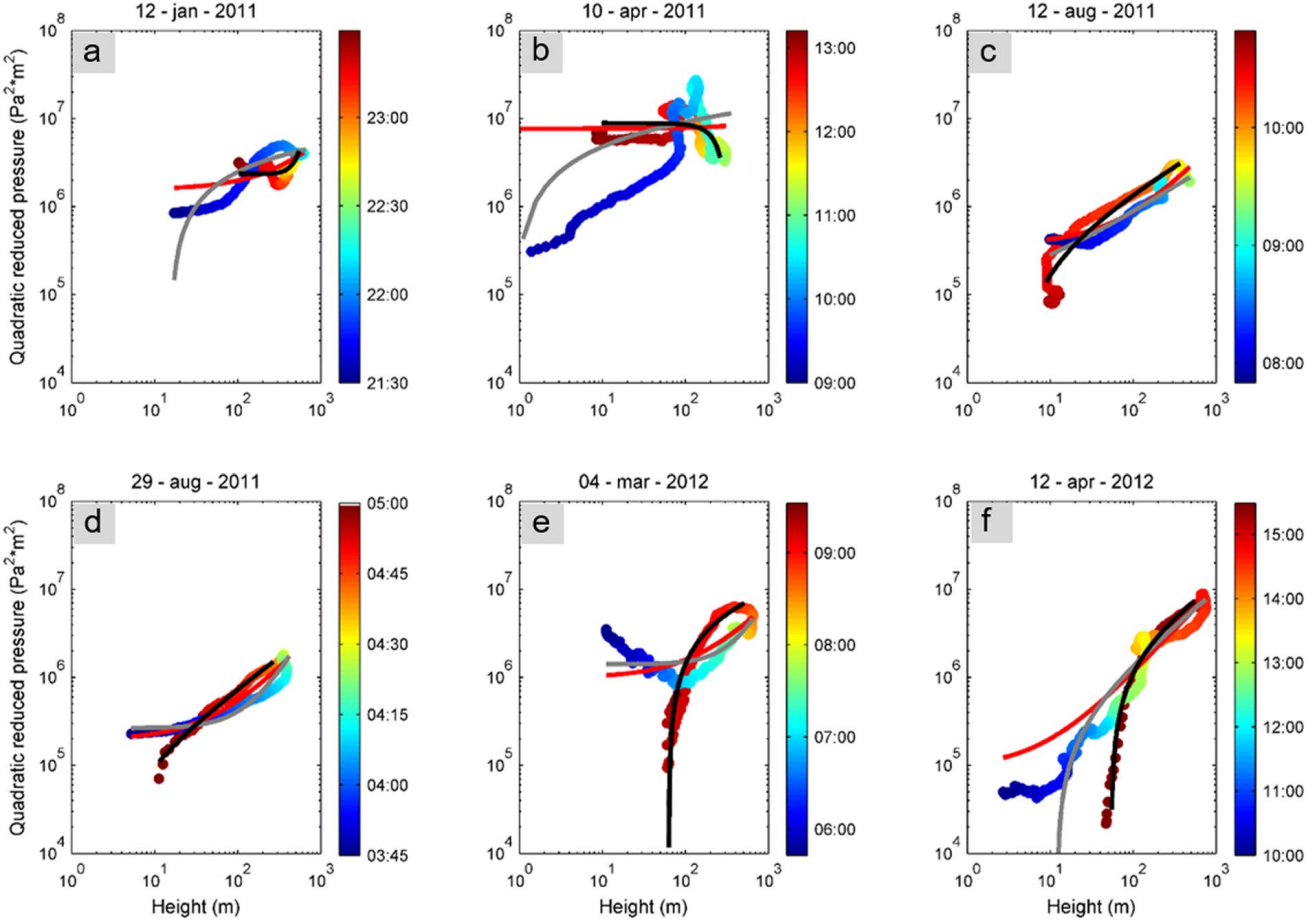
Infrasound-lava fountain height relationship. Relationship between quadratic reduced pressure, computed at EMFO, and lava fountain height as a function of time (in color scale) in the 0.5–5.0 Hz frequency band for each eruptive episode. Red curve represents the best fit assuming a linear relationship between the two variables; grey and black curves represent the best fit assuming a power law function, thus with the exponent (α in Eq. 2) variable for the waxing (solid grey line) and the waning (solid black line) phases of the eruptive episodes.

To investigate the relationship between volcanological parameters and seismo-acoustic signals, we plotted both quadratic reduced velocities and pressures versus lava fountain heights for the whole duration of each episode. We report the temporal information with the color scale, thus allowing us to explore the mutual time-dependent relationships between volcanological and geophysical parameters (Figs 3 and 4). Furthermore, with the aim of estimating volcanological parameters directly from geophysical data, we performed curve fitting of the data shown in Figs 3 and 4, assuming the following relation:

2 $$QR{A}_{s,i}=m\,\ast \,{H}_{lf}^{\alpha }+c$$

where *QRA*_*s*,*i*_ represents the quadratic reduced velocity and pressure, *H*_*lf*_ the lava fountain height, *α* the exponent describing the type of relation, *m* the scaling factor (corresponding with the slope in case of linear relationship, that is, *α = *1) and *c* an additional term. The *c* term was added due to the fact that both seismic and infrasonic amplitudes do not fall to zero during resting periods (characterized by *H*_*lf*_ = 0). To investigate such a relation, we assumed both a linear and a power law function and found the best fit curves.

## Results

Normalized acoustic data, seismic data, and lava fountain height are plotted in Fig. 2 for the six eruptive episodes analysed here. From a preliminary and qualitative comparison between the evolution of the volcanic activity and the seismic-infrasonic amplitude patterns, it is possible to observe a fairly similar behavior in all the analysed explosive episodes: (i) the resumption of explosive activity (initial Strombolian phase) is accompanied by a strengthening in both seismic and infrasonic amplitude; (ii) the climax of the eruptions (lava fountain phase) generally coincides with the maximum amplitudes in both seismic and infrasonic signals; (iii) the weakening of the explosive activity (and the successive end) is accompanied by a decrease in seismic and infrasonic amplitudes, reaching again the pre-eruptive levels. However, a more accurate analysis shows that a clear temporal shift often exists between quadratic reduced velocity, quadratic reduced pressure, and the fountain height envelopes. The different patterns observed in the quadratic reduced velocity and quadratic reduced pressure suggest that the contribution of coupling processes in the recorded seismic and infrasonic signals is negligible.

Figures 3 and 4 display the quadratic reduced velocities and pressures versus lava fountain heights, respectively. The relationships in these figures do not vary significantly depending on the frequency band, hence we decided to show results obtained in the wider 0.5–5.0 Hz frequency band. We also calculated an average value of the coefficient of determination (hereafter referred to as R^2^) among all the eruptive episodes for both power and linear fit, and it exhibited slightly higher values in this broad frequency band. Similarly, since EBEL and EMFO stations provided very comparable results (see Figures S2–S8 in the supplementary information), we show only results for EMFO station.

Examination of the reduced seismic velocities and acoustic pressures versus lava fountain heights shows, in general, a somewhat complex, time-dependent pattern. Maximum values of quadratic reduced velocity reached during each episode vary between about 0.07 and 0.22 m^4^/s^2^, with the lowest value obtained during the 12 January 2011 episode (Fig. 3). This episode was the first of 2011–2012 lava fountains and is characterized by a different behaviour with respect to the others. Indeed, focusing on this episode, the plot in Fig. 3a shows that the seismic signal has higher values with respect to the fountain heights during the waxing (initial) phase of the eruption as compared to the waning (ending) phase. This clear clockwise hysteresis pattern, as observed and termed by Fee *et al*.^16^, was observed at both stations. Similar clockwise hysteresis has also been observed between seismic river noise and water level (e.g.^38^). Conversely, all the other eruptive episodes (except for 4 March 2012 episode, showing a fairly complex trend) exhibit a counter-clockwise hysteresis pattern between seismic signal and lava fountain heights (Fig. 3). The acoustic quadratic reduced pressure shows maximum values in the range 1.8 × 10^6^ − 2.8 × 10^7^ Pa^2^ m^2^. The clockwise hysteresis pattern in the acoustic data vs fountain height for the 12 January eruption is fairly similar to the one observed for the seismic signals, while the counter-clockwise pattern observed in the seismic is only moderately apparent in the acoustic data (Fig. 4).

**Figure 5:**
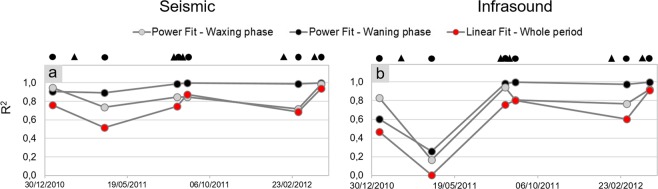
Best fit R^2^ values. Comparison of R^2^ values obtained for different types of relationships between quadratic reduced velocity and pressure (**a**,**b**, respectively) and lava fountain height for each of the eruptive episodes. Top black circles represent the eruptive episodes analysed in this study, while triangles represent the time occurrence of the previous eruptions.

**Figure 6:**
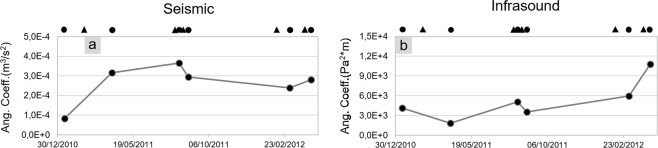
Angular coefficient values. Angular coefficient of the best fit assuming a linear relationship (α = 1 in Eq. 2) obtained between lava fountain height and quadratic reduced velocity (**a**) and pressure (**b**) for each of the eruptive episode. Top black circles represent the eruptive episodes analysed in this study, while triangles represent the time occurrence of the previous eruptions.

To quantitatively estimate the relationship between volcanological parameters and seismo-acoustic signals, linear regression between quadratic reduced velocities and pressures versus lava fountain heights was performed. Concerning the seismic regression, whose best-fit lines are reported with red colour in Fig. 3, the analysis shows good R^2^ values ranging between ~0.5 and ~1 (Fig. 5a). Similar regression line slopes (that is, the inclination angle to the x-axis, also called angular coefficient, numerically equivalent to the tangent of the angle and corresponding with *m* in Eq. (2)) exist among all the eruptive episodes (ranging between 2.4 and 3.6 × 10^−4^ m^4^/s^2^/m; Figs 3 and 6a), except for the first lava fountain (January 2011) (characterized by a lower value of 8.3 × 10^−5^ m^4^/s^2^/m; Figs 3a and 6a). The angular coefficients and R^2^ are more variable for the acoustic data (Figs 4, 5b and 6b).

In the light of the similarity among the slopes obtained for the seismic linear regressions, we made lava fountain height estimations in all the six investigated explosive episodes based on the quadratic reduced velocity and the angular coefficient (taking the median value). The average and standard deviation values of the errors, computed as the absolute value of the difference between observed and estimated lava fountain heights, for the six explosive episodes were equal to 110 and 80 m, respectively.

In order to further examine these aforementioned patterns and to investigate the relationships between lava fountain heights and seismic and acoustic amplitudes in the different phases of the eruption, we performed curve fitting analysis assuming a power law function to define the exponent better fitting the relationship between the time series (solid grey and black lines in Figs 3 and 4). In particular, we split the eruptive episodes into a waxing phase (from the beginning to the maximum value of the lava fountain height) and a waning one (from the maximum value of height until the end).

**Figure 7:**
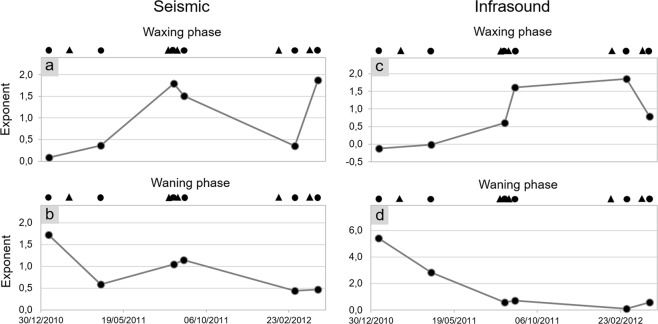
Best-fit exponents. Best-fit exponents (α in Eq. 2) of the power law fitting the quadratic reduced velocity and lava fountain height during the waxing and waning (**a**,**b**, respectively) phases of the analysed eruptive episodes. Best-fit exponents (α in the Eq. 2) of the power law fitting the quadratic reduced pressure and lava fountain height during the waxing and waning (**c**,**d**, respectively) phases of the analysed eruptive episodes. Top black circles represent the eruptive episodes analysed in this study, while triangles represent the time occurrence of the previous eruptions.

**Figure 8:**
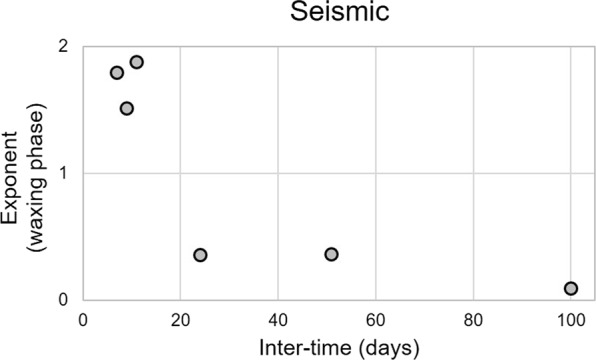
Exponent-inter-time relationship. Best-fit exponents assuming a power law function between quadratic reduced velocity and lava fountain height during the waxing phase plotted versus the inter-eruption time (in days) between the analysed lava fountain and the previous one.

Regarding the relationship between quadratic reduced velocity and lava fountain height during the waxing phase, we found exponent values (α in Eq. 2) ranging between 0.1 and 1.9 (Fig. 7a). In particular, among the eruptive episodes, three are characterized by an exponent lower than 1, that is seismic energy increases at a greater rate with respect to height (solid grey lines in Figs 3a,b,e and 7a), while for the others the exponent is greater than 1, hence seismic energy increases at a lower rate (solid grey lines in Figs 3c,d,f and 7a). It is also noteworthy that eruptive episodes with exponent greater than 1 are those characterized by a short inter-eruptive time, that is, occurred just a few days after the previous lava fountain (Fig. 8). Regarding the goodness of fit between seismic data and lava fountain heights, results show fairly good R^2^ values ranging from ~0.7 to ~1.0 (Fig. 5a).

We also found significant differences in terms of the exponents during the waning phases (solid black line in Fig. 3): three of them show exponents lower than 1; for the 12 and 29 August 2011 episodes, the relationship is almost linear (α~1); for the 12 January 2011 α is higher than 1 (Fig. 7b). During this phase, R^2^ values are higher, ranging from ~0.9 to ~1.0 (Fig. 5a).

We found much more variable relationships for the quadratic reduced pressure versus lava fountain heights (solid grey and black lines in Fig. 4), despite the good quality of the fit (R^2^ values between ~0.6 and ~1.0, except for one episode, Fig. 5b). The acoustic exponents vary in a wide range, between −0.1 and 5. (Fig. 7c,d).

## Discussion

Despite the significant potential impact of explosive volcanic eruptions, accurate, 24-7, real-time characterization of ongoing eruptive phenomena is impossible, yet. In order to achieve real-time eruption plume and fountain characterization, we attempted to derive a scaling factor by quantitatively exploring the relationship between seismic and acoustic amplitude and lava fountain height. For the seismic data, the angular coefficient obtained in the case of linear regression in 5 out of 6 eruptive episodes is relatively stable, as well as the fairly high values of the R^2^ parameter (Figs 6a and 5a respectively). On the basis of the quadratic reduced velocity and such a steady angular coefficient, it was possible to perform lava fountain height estimations with average error of 110 m. This can be considered as a very promising step towards the aforementioned goal. Indeed, on the basis of this result it is possible to make inferences on the fountain height by quadratic reduced velocity values. Once the fountain height is known, the mean fluid exit velocity at the vent (*U*) can be calculated by applying the following equation (e.g.^39^):

3 $$U=\sqrt{2g{H}_{lf}}$$

where *g* is the gravity acceleration. If the velocity is known, then realistic estimates of mass eruption rate and plume height/extent can be made.

Although assuming a linear relationship between quadratic reduced velocity and lava fountain height permits a straightforward derivation of eruption source parameters from seismic data, we find that the best fit is actually obtained with distinct power laws described by exponents α of Eq. (2) ranging from 0.1 to 1.9. On the basis of the Eqs (2) and (3), the relationship linking quadratic reduced velocity and fluid exit velocity has to be characterized by an exponent equal to twice α:

4 $$QR{A}_{s}-c\propto {H}_{lf}^{\alpha }\propto {U}^{2\alpha }$$

where the first term “*QRA*_*s*_ *– c*” can be considered as the increase of quadratic reduced velocity during eruptive periods with respect to the non-eruptive intervals (characterized by quadratic reduced velocity equal to *c*, see Eq. (2)). Hence, on the basis of the results of this work, the exponent values describing the relationship between quadratic reduced seismic velocity and fluid exit velocity should be 0.2-3.8, that is a range which in the upper bound is similar to that highlighted by Ichihara^11^, who summarized the different power law relations obtained from various datasets and models by previous works. In particular, based on observations for different eruptions from the literature, McNutt^40^ found a relationship between reduced displacement and plume height with an exponent of 1.8. This corresponds to a value of 0.9 in the relationship between seismic energy versus magma discharge rate that is proportional to the fluid exit velocity^11^. On the other hand, Prejean and Brodsky^18^ obtained a power law with exponent equal to 4 for the relationship between seismic energy versus magma discharge rate. Ichihara^11^ observed linear relationships between seismic energy, infrasonic energy and magma discharge rate during the three sub-Plinian events of the 2011 Shinmoe-dake eruption she studied, and in particular during the quasi-stable or slowly growing stages of the events. Based on these findings she hypothesized a common seismic-infrasonic source, located in the subsurface, and proposed an explanation for the above-mentioned stage of the events. Such a quasi-linear relationship also agrees with the linear scaling between seismic energy and eruption rate inferred by Haney *et al*.^20^. It has to be said that we obtained similar exponent values only in two out of six analysed lava fountains, indeed in the second and the fifth episode, the exponents obtained for the power law quadratic reduced velocity vs fluid exit velocity during the waxing phase of the lava fountain is 0.8 (twice the exponent values in the quadratic reduced velocity vs lava fountain height), hence suggesting a roughly linear relationship for these episodes. However, the exponents computed during the other four episodes strongly deviate from the linear scaling, thus suggesting much more complex and time-variable processes with respect to steady state flow condition.

The power law exponent variability found here appears to be related to the time interval from the previous eruption (Fig. 8). In particular, we observe that the longer the time from the previous eruption, the lower the exponent; that is seismic amplitude squared increases at a greater rate with respect to the lava fountain height. This suggests that the conditions of the uppermost part of the plumbing system, in terms of presence of plug obstructing the vent and/or magma features (gas content, viscosity, and so on), somehow affect such a relationship. The dependence of such an exponent from the conditions of the plumbing system has also been inferred by Spina *et al*.^41^ by laboratory experiments. In particular, they observed how increase in viscosity of the analogue magma filling the conduit leads to a decrease in the exponent of the power law connecting seismic squared amplitudes and air flow rate. This link derives from friction, affecting the seismic energy release, that is a function of several parameters including the kinematic viscosity (e.g.^42,43^). On the basis of these experimental results, it is possible to qualitatively interpret our observations as follows: the longer the time interval from the previous eruptive episode, the longer the time magma resides in the shallow plumbing system. Degassing and crystallization then lead to increased viscosity. Therefore, the exponent should decrease with increasing time interval from the previous eruptive episode.

Furthermore, it was highlighted how only the 12 January 2011 lava fountain shows a clear clockwise hysteresis. According to Fee *et al*.^44^, the higher level of seismic noise during the waxing phase could be due to the fact that the upper portion of plumbing system at the beginning of the eruption was in a densely packed state, plugged with degassed magma. The high velocity of particle and gas during the eruption eroded the walls of the uppermost portion of conduit, thus diminishing the energy transfer toward the rocks and then the generation of seismic energy during the waning phase. This model is supported by the fact that this lava fountain was the first episode of the 2011-2017 eruptive sequence, as well as the first eruption taking place at NSEC.

Variability in the relationship between volcanic tremor amplitude and lava fountain height was also observed by Eaton *et al*.^21^, who analysed the 1959 Kilauea Iki eruption. Similar to what we observed during the 12 January 2011 episode, Eaton *et al*.^21^ reported a decrease in the ratio between volcanic tremor amplitude vs lava fountain height (and a decrease in seismic efficiency) during the course of the eruption. According to these authors, one of the factors mostly affecting such a relationship is the geometry of the vent ejecting the lava fountain: a dike, with a large surface-area-to-volume ratio, is much more efficient in radiating elastic energy than a central conduit. In addition, they stated that magma temperature (influencing the viscosity) and gas content and composition play also an important role in determining the lava fountain behavior, and its relationship to volcanic tremor.

These lines of evidence suggest the seismic tremor source is closely linked to the state of the upper conduit prior to eruption and seismic “efficiency” changes during the eruption. Eruption monitoring and source characterization from geophysical data should take these factors into account.

Also, as regards the 12 January 2011 lava fountain, this eruption was characterized by lower quadratic reduced velocity values with respect to the other ones (Fig. 3). As demonstrated in literature^40,45^, volcanic tremor features (amplitude and spectral content) likely depend on the geometry and size of the volcanic conduit, as well as the degree of explosivity and the style of the eruption. Furthermore, McNutt and Nishimura^19^, studying the correlation between volcanic tremor and the cross‐sectional area of the conduit, found that the tremor reduced displacement linearly increases with crater radius. Therefore, seeing as all the paroxysmal events examined here exhibited the same eruptive style, the higher volcanic tremor energy, observed during all the analysed lava fountain episodes with respect to the first one, could be due to an enlargement of volcanic conduit/vent. Indeed, the 12 January eruptive episode is considered the first of the numerous eruptive episodes that morphologically built NSEC as an independent cone^26^.

Concerning the quadratic reduced pressures versus lava fountain height relationship, more variable results were obtained with respect to the quadratic reduced velocity in terms of both angular coefficients of the linear regression and power law exponents (Figs 6b and 7c,d respectively). Such greater variability could be related to a more pronounced directionality of the acoustic radiation with respect to seismic one. Indeed, although also seismic sources can be anisotropic (e.g.^30^), scattering phenomena, much more effective on seismic waves propagating within a volcanic edifice than on acoustic waves for short propagation distances (e.g.^46^), tends to make seismic energy radiation more uniformly distributed in the medium surrounding the source.

## Concluding remarks

Quantitative investigation of the relationship between temporal evolution of lava fountain heights and time series of seismic and acoustic amplitude squared during eruptive episodes at Mt. Etna led to the following findings:

- by performing a linear regression between quadratic reduced velocity and lava fountain height, fairly constant values of the scaling factor were found in 5 out of 6 eruptive episodes. This can be considered as a promising step to derive eruption source parameters (such as fountain height, jet velocity, etc) from geophysical data;
- the exponents determined for the power law fit linking quadratic reduced velocity and lava fountain height mostly fall in the range defined in the literature (e.g.^11^). The exponent value is likely influenced by the time interval from the previous eruption, suggesting that the conditions of the uppermost part of the plumbing system (in terms of the presence of a plug obstructing the vent and/or magma features) play a key role in the seismic (and perhaps acoustic) energy generation during the eruptions;
- the first lava fountain of the 2011–2017 eruptive sequence showed a clear clockwise hysteresis pattern, with higher level of seismic and acoustic amplitude during the waxing phase. This was probably associated with the erosion of the walls of the uppermost portion of conduit, that diminished the energy transfer toward the rocks during the waning phase.

## Supplementary information

Supplementary Information
