# Supplementary material for: Unravelling the links between seismo-acoustic signals and eruptive parameters: Etna lava fountain case study
Source: Sci Rep. 2019 Nov 11;9:16417. doi: 10.1038/s41598-019-52576-w (PMC6848099; doi:10.1038/s41598-019-52576-w)
Supplement: Supplementary file 1 — Supplementary Information [file 41598_2019_52576_MOESM1_ESM.pdf]

Supplementary Information for:

Unravelling the links between seismo-acoustic signals and eruptive parameters: Etna lava fountain case study

Mariangela Sciotto[1], Andrea Cannata[1,2], Michele Prestifilippo[1], Simona Scollo[1], David Fee[3], Eugenio Privitera[1]

1. Istituto Nazionale di Geofisica e Vulcanologia, Osservatorio Etneo, Piazza Roma 2, 95125, Catania, Italy
2. Università degli Studi di Catania, Dipartimento di Scienze Biologiche, Geologiche e Ambientali – Sezione di Scienze della Terra, Corso Italia 57, I-95129, Catania, Italy
3. Alaska Volcano Observatory, University of Alaska Fairbanks Geophysical Institute, Fairbanks, AK 99775, USA

Paper submitted to Scientific Reports

Corresponding author: Mariangela Sciotto: mariangela.sciotto@ingv.it

Supplementary material:

1. Eruptive Activity

Below we briefly describe each fountaining episode:

1.1. *The 12 January 2011 lava fountain*

The lava fountain of 12 January 2011 was the first event that occurred at the pit crater located at the base of SEC (now NSEC), and mainly for this reason is one of the most studied events. The chronology of this event is described in detail by Calvari *et al.*[1]. The eruption began on 11 January 2011 and was visible from the video network only after 17:50 UTC (all times reported are UTC) due to bad weather conditions. Strombolian activity was variable in intensity and a lava flow formed at 20:20 from the lower rim of the pit crater. Strombolian activity then increased in frequency after 21:00 while lava fountains formed at 21:50[2] and produced a 9 km height a.s.l (above sea level) column[3]. The tephra fallout covered the south west volcano flanks and was sampled up to 100 km from the summit craters[4]. The total mass and the whole total grain-size estimated from the deposit was $1.5 \pm 0.4 \times 10^8$ kg[4]. The lava fountain ended at 23:50 while the eruptive episode at 02:00 of 13 January 2011[2].

1.2. The 10 April 2011 lava fountain

The lava fountain of 10 April 2011 was the third paroxysmal event in 2011. Strombolian activity began early in the morning two days before while a lava flow began at 17:55 on 9 April[2] and was directed toward the SE for about 1.5 km[5]. The lava fountain started at about 08:00 and lasted more than 5 hours[2] with intensity that changed in time while the eruptive episode ended after 14:05. The maximum column height seen by satellite reached 5.5 km a.s.l.[3] and produced tephra fallout on the SE flank. On the base of the analysis of geophysical data (tilt, seismic tremor, gravity, gas and satellite thermal data), Bonaccorso *et al.*[5] estimated a total erupted dense rock equivalent (DRE) volume of about 1×10^6 m^3.

1.3. The 12 August 2011 lava fountain

Eruptive activity began early in the morning on 11 August 2011 with sporadic ash emission that intensified to Strombolian activity at 05:30[2]. A lava flow followed and preceded the paroxysmal phase between 08:30 and 10:00[2]. The peak of the lava fountain activity occurred about 09:30 and the maximum column height was 9.5 km a.s.l.[6]. The activity was retrieved by a monitoring calibrated camera that was able to track the temporal evolution of the fountain and plume[2]. After 10:00 the activity decreased and ended at 11:00.

1.4. The 29 August 2011 lava fountain

The 29 August 2011 eruption was the twelfth episode in 2011 and began with weak Strombolian activity on 28 August at 18:00[2]. The day after, early in the morning, there was a new lava flow that preceded the lava fountain by only 1 hour. The paroxysmal phase occurred between 04:05 and 04:40 and produced an eruption column that unfortunately was not retrieved by satellite due to the bad weather condition[3] but caused a tephra fallout deposit toward the southeast direction. The eruptive activity ended at 05:15 of the same day[2]. Noteworthy is that at 04:20 there was an opening of a new fracture in the southeastern flank of the NSEC.

1.5. *The 4 March 2012 lava fountain*

The 4 March lava fountain was the third paroxysmal event in 2012 and occurred between 07:30 and 09:32[2]. The activity was preceded by small ash emissions the month before and by a lava flow starting at 06:00 of the same day. The activity was then characterized by the formation of a small pyroclastic flow at about 07:50 and, at the same time, by a lava flow from a new eruptive vent opened on the upper southwestern flank of the NSEC (http://www.ct.ingv.it/it/component/content/article/11-notizie/news/504). The intensity of this activity was high with the formation of an eruption column of about 8 km a.s.l. and copious tephra fallout that covered the northeast flanks. The activity ended at 09:32 of the same day[2].

1.6. The 12 April 2012 lava fountain

The 12 April 2012 lava fountain was the sixth event that occurred in 2012. Strombolian activity started in the evening of the day before, then increased in frequency and transitioned to lava fountain activity at 14:30. The paroxysmal phase lasted up to 15:15 while the eruptive activity ended at 16:00[2]. During this event, the interaction of the lava flow and snow formed a vapor column that rose 1 km high above the crater. The eruption column was not retrieved by satellite due to poor weather conditions but was detected by the calibrated camera recording in the visible-band[7] and reached a maximum height of 8.5 km a.s.l. Ash and lapilli fell on the east volcano flanks.

Supplementary figures:

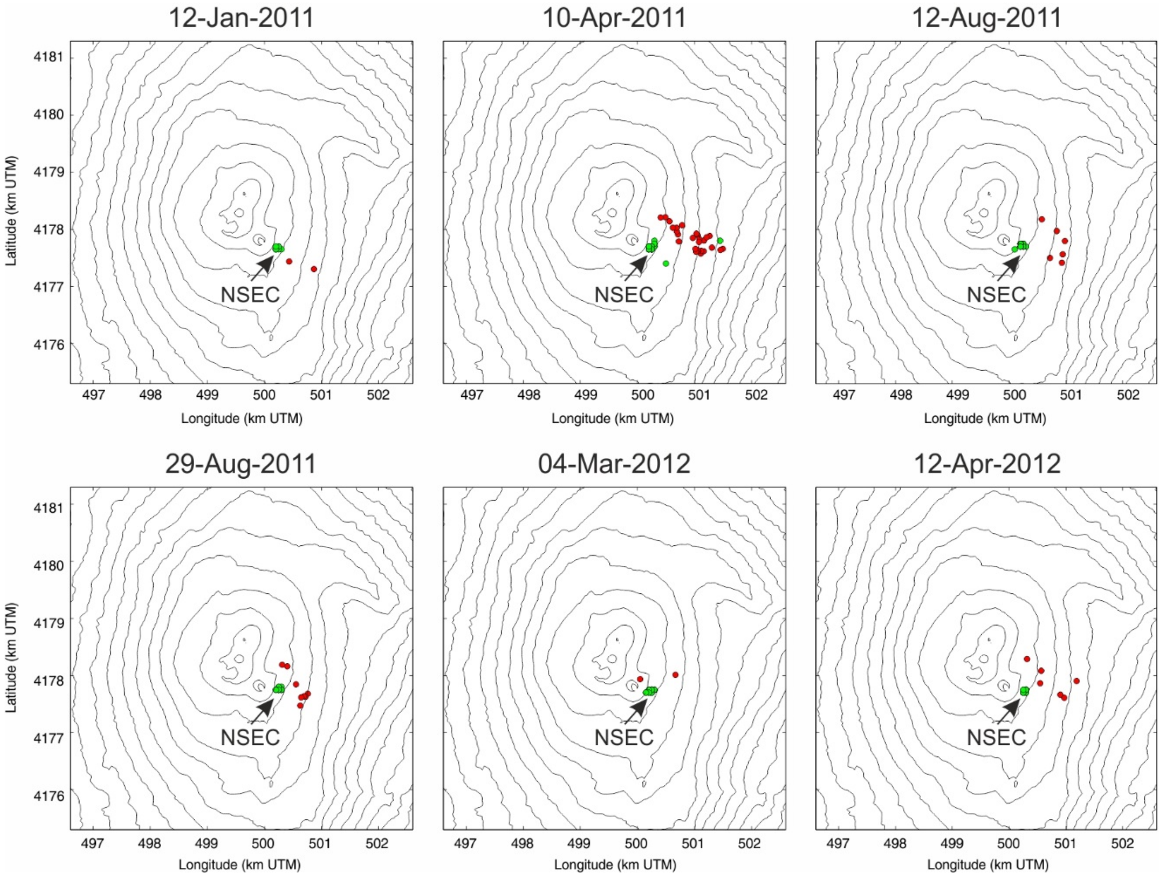

Figure S1. Infrasonic and seismic source locations. Digital elevation model of Mt. Etna, showing the locations of the infrasonic sources (green dots) and volcanic tremor centroids (red dots) during the six considered lava fountain episodes.

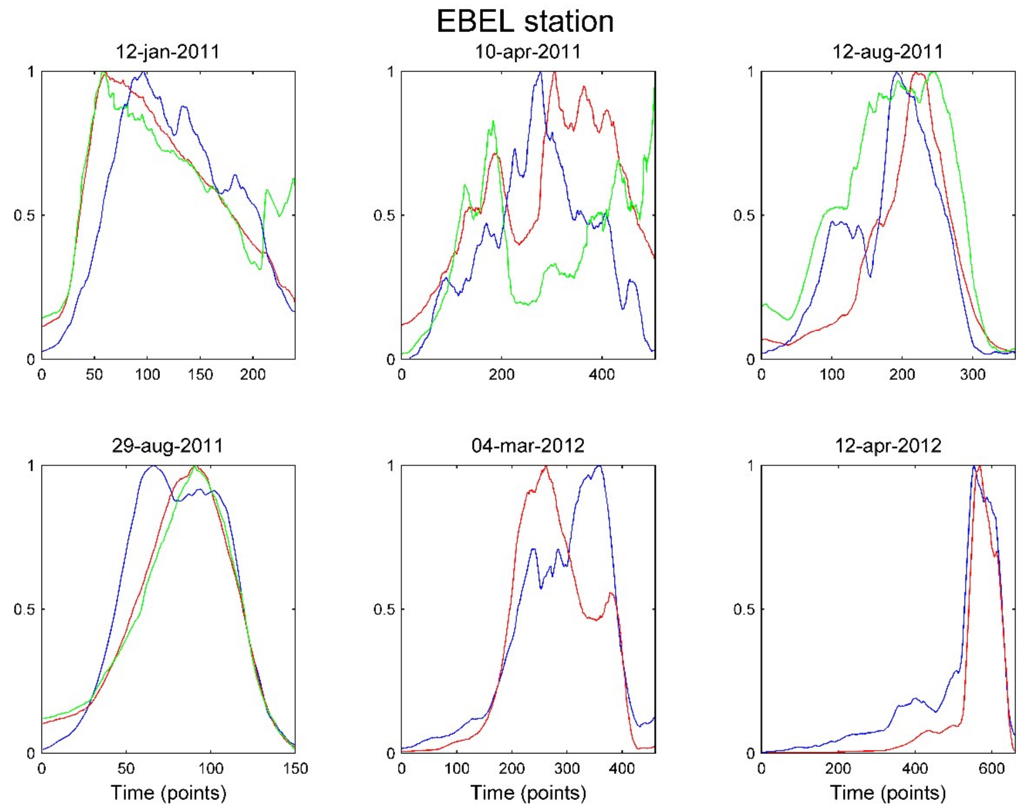

Figure S2. Multiparametric time series. Normalized quadratic reduced velocity (red line) and pressure (green line) envelope in the 0.5 – 5.0 Hz frequency band, computed at EBEL, and lava fountain height values (blue line) for each eruptive episode. As regarding the 4 March and 12 April 2012 eruptive episodes, we do not show acoustic data since EBEL infrasonic station did not properly work.

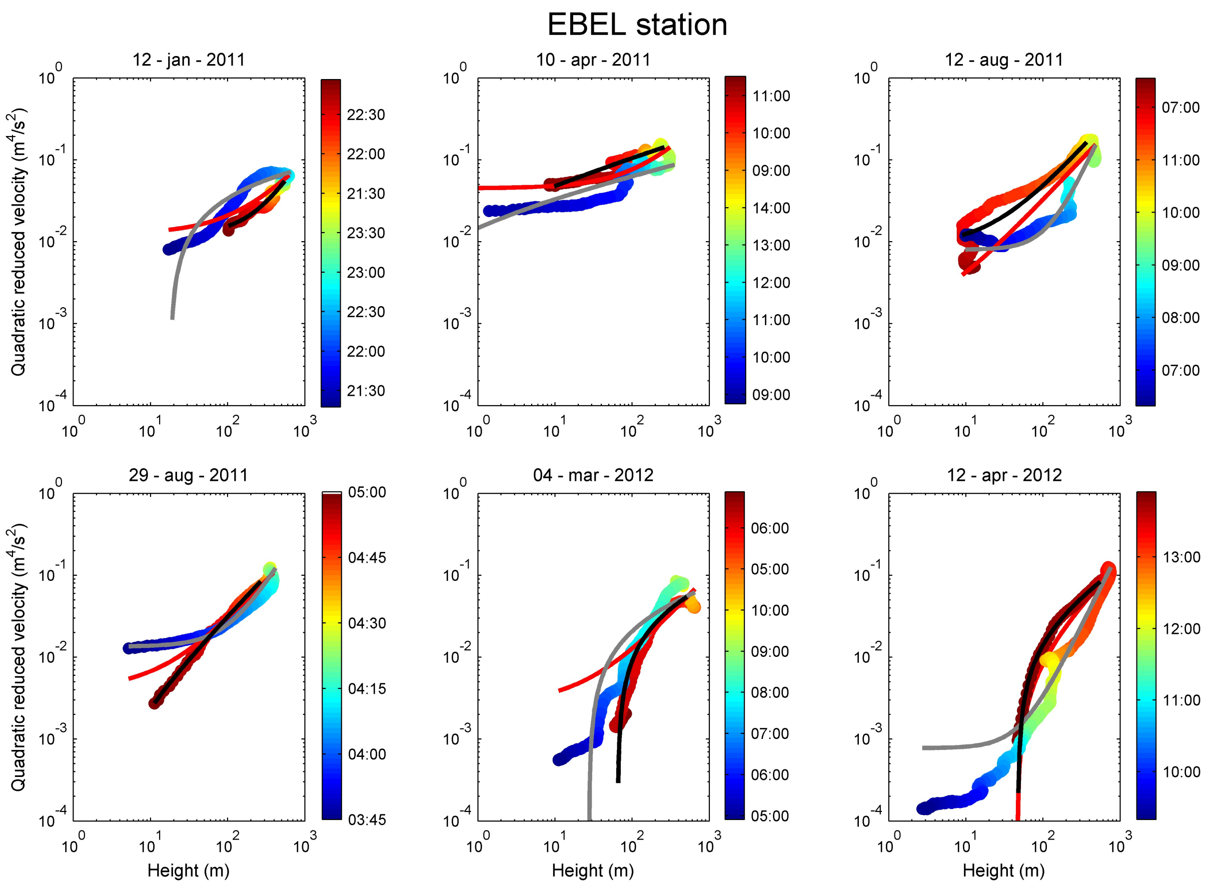

Figure S3. Seismic-lava fountain height relationship. Relationship between quadratic reduced velocity, computed at EBEL, and lava fountain height as a function of time (in color scale) in the 0.5 – 5.0 Hz frequency band for each eruptive episode. Red curve represents the best fit line assuming a linear relationship between the two variables; grey and black curves represent the best fit assuming a power law function, thus with the exponent (α in equation 2) variable for the waxing (solid grey line) and the waning (solid black line) phases of the eruptive episode.

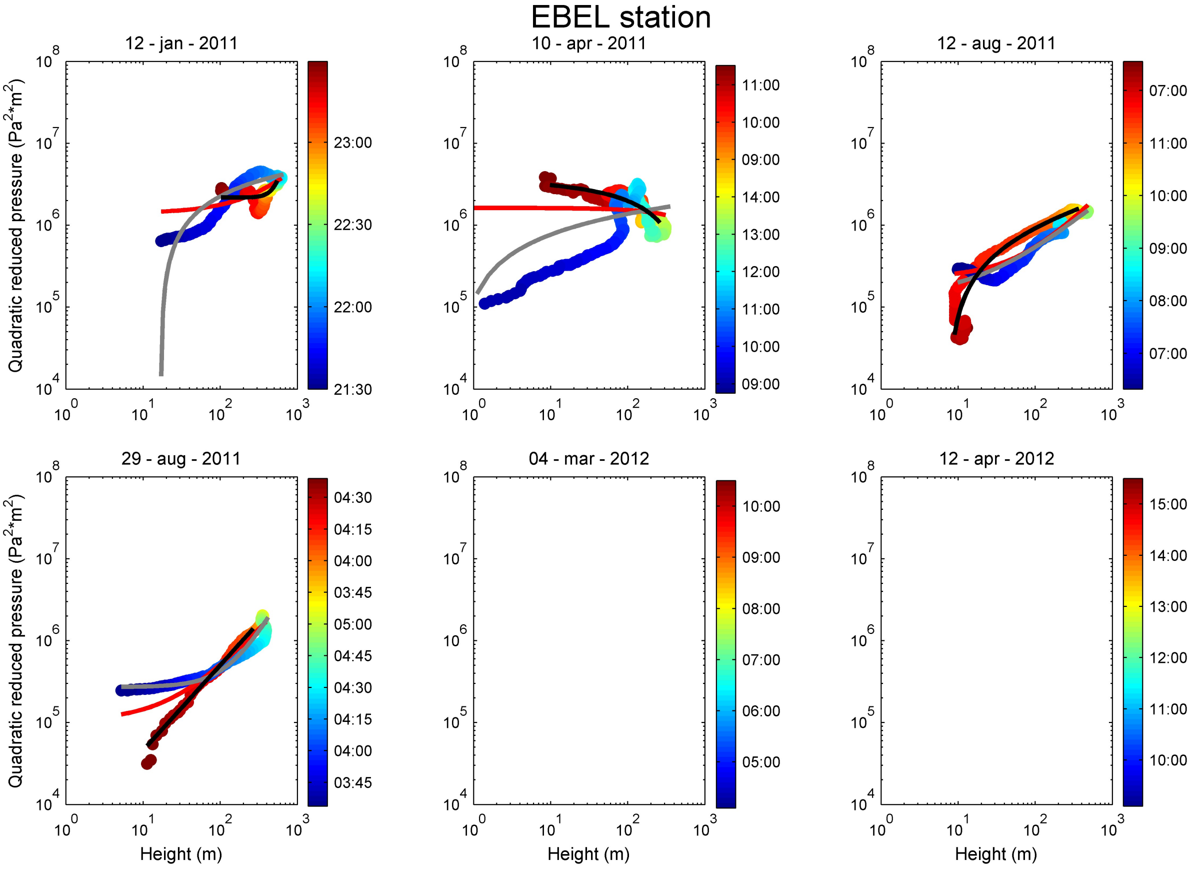

Figure S4. Infrasound-lava fountain height relationship. Relationship between quadratic reduced pressure, computed at EBEL, and lava fountain height as a function of time (in color scale) in the 0.5 – 5.0 Hz frequency band for each eruptive episode. Red curve represents the best fit line assuming a linear relationship between the two variables; grey and black curves represent the best fit assuming a power law function, thus with the exponent (α in equation 2) variable for the waxing (solid grey line) and the waning (solid black line) phases of the eruptive episodes. As regarding the 4 March and 12 April 2012 eruptive episodes, we do not show acoustic data since EBEL infrasonic station did not properly work.

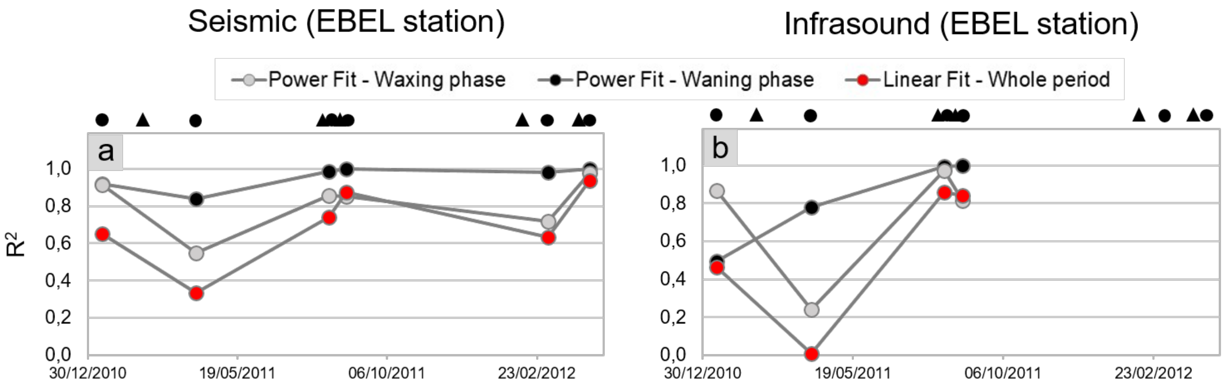

Figure S5. Best fit R^2 values. Comparison of R^2 values obtained for different types of relationships between quadratic reduced velocity and pressure (**a** and **b**, respectively) and lava fountain height for each of the eruptive episodes. Top black circles represent the eruptive episodes analysed in this study, while triangles represent the time occurrence of the previous eruptions. As regarding the 4 March and 12 April 2012 eruptive episodes, we do not show acoustic data since EBEL infrasonic station did not properly work.

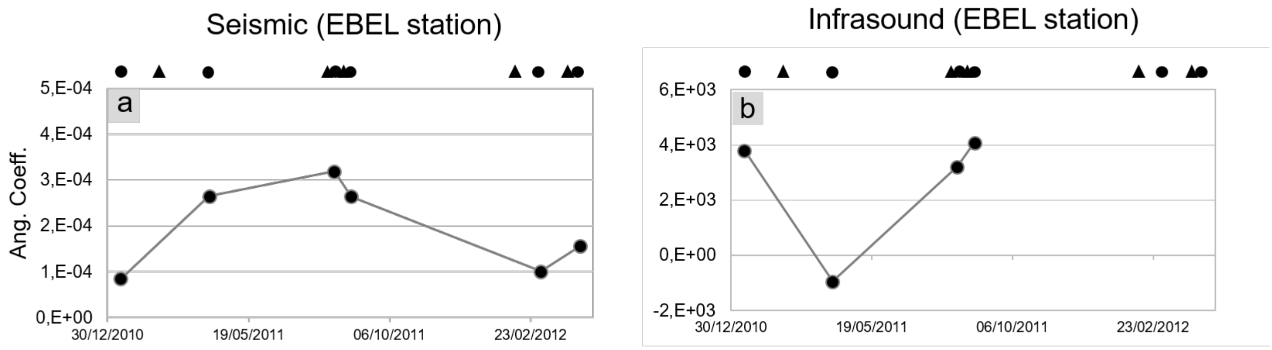

Figure S6. Angular coefficient values Angular coefficient of the best fit assuming a linear relationship (α=1 in equation 2) obtained between lava fountain height and quadratic reduced velocity (**a**) and pressure (**b**) for each of the eruptive episode. Top black circles represent the eruptive episodes analysed in this study, while triangles represent the time occurrence of the previous eruptions. As regarding the 4 March and 12 April 2012 eruptive episodes, we do not show acoustic data since EBEL infrasonic station did not properly work.

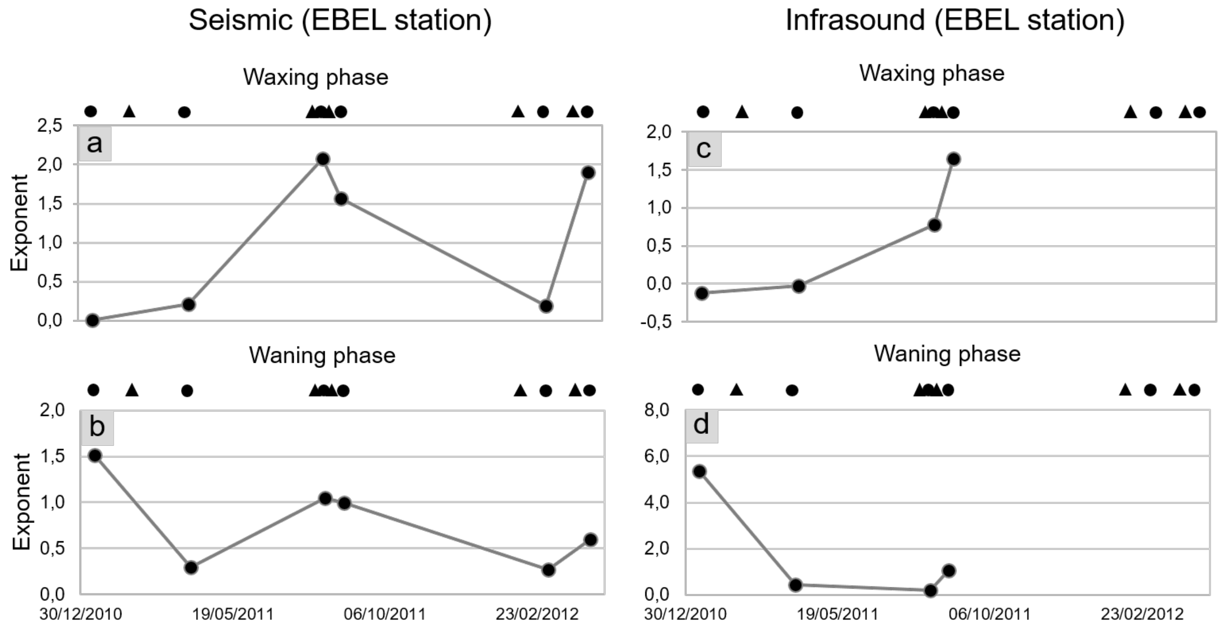

Figure S7. Best-fit exponents. Best-fit exponents (α in equation 2) of the power law fitting the quadratic reduced velocity and lava fountain height during the waxing and waning (**a** and **b**, respectively) phases of the analysed eruptive episodes. Best-fit exponents (α in the equation 2) of the power law fitting the quadratic reduced pressure and lava fountain height during the waxing and waning (**c** and **d**, respectively) phases of the analysed eruptive episodes. Top black circles represent the eruptive episodes analysed in this study, while triangles represent the time occurrence of the previous eruptions. As regarding the 4 March and 12 April 2012 eruptive episodes, we do not show acoustic data since EBEL infrasonic station did not properly work.

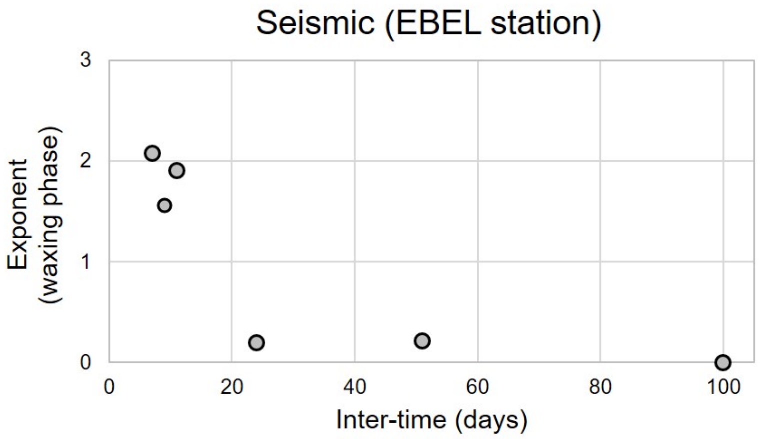

Figure S8. Exponent-inter-time relationship. Best-fit exponents assuming a power law function between quadratic reduced velocity and lava fountain height during the waxing phase plotted versus the inter-eruption time (in days) between the analysed lava fountain and the previous one.

Supplementary references

1. Calvari, S., *et al*. An unloading foam model to constrain Etna's 11–13 January 2011 lava fountaining episode. *J. Geophys. Res*. **116**, B11207, doi:10.1029/2011JB008407 (2011).
2. Behncke, B., *et al.* The 2011–2012 summit activity of Mount Etna: Birth, growth and products of the new SE crater. *J. Volcanol. Geotherm. Res.* **270**, 10-21 (2014).
3. Corradini, S., *et al*. Proximal monitoring of the 2011-2015 Etna lava fountains using MSG-SEVIRI data. *Geosciences* **8** (140), doi: 10.3390/geosciences8040140 (2018).
4. Andronico, D., Scollo, S., Cristaldi, A. & Lo Castro, M. D. Representivity of incompletely sampled fall deposits in estimating eruption source parameters: A test using the 12–13 January 2011 lava fountain deposit from Mt. Etna volcano, Italy. *Bull. Volcanol.* **76**, doi:10.1007/s00445-014-0861-3 (2014).
5. Bonaccorso, A., *et al*. Dynamics of a lava fountain revealed by geophysical, geochemical and thermal satellite measurements: The case of the 10 April 2011 Mt Etna eruption. *Geophys. Res. Lett.* **38**, L24307, doi:10.1029/2011GL049637 (2011).
6. Scollo, S. *et al*. Volcanic ash concentration during the 12 August 2011 Etna eruption. *Geophys. Res. Lett.* **42**, 2634–2641, https://doi.org/10.1002/2015GL063027 (2015).
7. Scollo, S. *et al*. Eruption column height estimation of the 2011–2013 Etna lava fountains. *Ann. Geophys*. **57**, 0214, https://doi.org/10.4401/ag-6396 (2014).
